# Association of Perfluorooctanoic Acid (PFOA) and Perfluorooctane Sulfonate (PFOS) with Uric Acid among Adults with Elevated Community Exposure to PFOA

**DOI:** 10.1289/ehp.0900940

**Published:** 2009-10-22

**Authors:** Kyle Steenland, Sarah Tinker, Anoop Shankar, Alan Ducatman

**Affiliations:** 1 Rollins School of Public Health, Emory University, Atlanta, Georgia, USA; 2 Department of Community Medicine, West Virginia University School of Medicine, Morgantown, West Virginia, USA

**Keywords:** PFOA, PFOS, uric acid

## Abstract

**Background:**

Perfluorooctanoic acid (PFOA) and perfluorooctane sulfonate (PFOS) are compounds that do not occur in nature, have been widely used since World War II, and persist indefinitely in most environments. Median serum levels in the United States are 4 ng/mL for PFOA and 21 ng/mL for PFOS. PFOA has been associated with elevated uric acid in two studies of chemical workers. Uric acid is a risk factor for hypertension and possibly other cardiovascular outcomes.

**Methods:**

We conducted a cross-sectional study of PFOA and PFOS and uric acid among 54,951 adult community residents in Ohio and West Virginia, who lived or worked in six water districts contaminated with PFOA from a chemical plant. Analyses were conducted by linear and logistic regression, adjusted for confounders.

**Results:**

Both PFOA and PFOS were significantly associated with uric acid. An increase of 0.2–0.3 mg/dL uric acid was associated with an increase from the lowest to highest decile of either PFOA or PFOS. Hyperuricemia risk increased modestly with increasing PFOA; the odds ratios by quintile of PFOA were 1.00, 1.33 [95% confidence interval (CI), 1.24–1.43], 1.35 (95% CI, 1.26–1.45), 1.47 (95% CI, 1.37–1.58), and 1.47 (95% CI, 1.37–1.58; test for trend, *p* < 0.0001). We saw a less steep trend for PFOS. Inclusion of both correlated fluorocarbons in the model indicated PFOA was a more important predictor than was PFOS.

**Conclusion:**

Higher serum levels of PFOA were associated with a higher prevalence of hyperuricemia, but the limitations of cross-sectional data and the possibility of noncausal mechanisms prohibit conclusions regarding causality.

Perfluorooctanoic acid (PFOA) and perfluorooctane sulfonate (PFOS) are perfluorinated compounds that have been found in the blood of virtually all Americans tested during the last decade (Calafat et al. 2007). They do not occur naturally but were introduced in the environment after World War II.

PFOA is used as a polymerization aid in the manufacture of several types of fluoropolymers, which have been used in a wide variety of industrial and consumer products, such as Teflon and Gore-Tex. PFOA does not break down in most environments. The half-life of PFOA in humans is estimated to be 3.8 years [arithmetic mean; 95% confidence interval (CI), 3.1–4.4 years] (Olsen et al. 2007). The median level in the U.S. population was 4 ng/mL in 2003 (Calafat et al. 2007). The origins of long-chain per-fluorocarbon exposures stem from manufacture or use of industrial products, yet the routes of exposure and specific origins are rarely clear. PFOA is also widespread in the serum of inhabitants of many other countries (Lau et al. 2007).

PFOA has been found to be significantly associated with elevated uric acid in two cross-sectional studies of chemical workers (*n* = 160 and *n* = 1,024) (Costa et al. 2009; Sakr et al. 2007a). There is also evidence in the literature for an association of PFOA with cholesterol and diabetes in humans. A positive correlation of PFOA with cholesterol was observed in six occupational studies (Costa et al. 2009; Olsen and Zobel 2007; Olsen et al. 2000, 2003; Sakr et al. 2007a, 2007b), and two community studies (Emmett et al. 2006; Steenland et al. 2009b), although in one community study and two of occupational studies the relationship was not statistically significant. PFOA exposure was observed to be associated with a 2-fold increase in diabetes mortality in one cohort study of highly exposed workers compared with nonexposed workers, although no association was seen in another cross-sectional study of diabetes prevalence (Leonard et al. 2008; MacNeil et al. 2009). In addition, PFOA has been associated with a number of outcomes in animal data, particularly tumors and neonatal loss (Lau et al. 2007; U.S. EPA 2005).

PFOS is another perfluorocarbon that is widespread in the serum of U.S. residents, with a median serum level of 21 ng/mL in 2003 (Calafat et al. 2007). Although PFOA is found at much higher levels among our study subjects than in the U.S. general population, PFOS levels were similar to U.S. general population levels, suggesting that the nearby industrial facility was not a significant source of PFOS. Until recently, PFOS was used in the manufacture of Scotchgard among other products. Its half-life in humans has been estimated at 5.4 years (arithmetic mean; 95% CI, 3.9–6.9 years) (Olsen et al. 2007). In one cross-sectional study of PFOS and cholesterol, a statistically significant positive association was observed among workers at one plant, but not at a second plant (Olsen et al. 1999). There are no data of which we are aware regarding an association of PFOS and uric acid.

Uric acid is a natural product of purine metabolism and has both oxidant and antioxidant properties. Considerable epidemiologic evidence exists from longitudinal studies, supported by animal evidence, that elevated uric acid is a risk factor for hypertension (Feig et al. 2006; Mellen et al. 2006; Shankar et al. 2006; Sundström et al. 2005). A recent randomized trial found that lowering uric acid resulted in lowering blood pressure in adolescents (Feig et al. 2008). On the other hand, there is debate about whether uric acid is a predictor of cardiovascular disease, independent of other known risk factors, and independent of its role as a marker of kidney disease (Fang and Alderman 2000; Hakoda et al. 2005; Johnson et al. 2003; Shankar et al. 2006; Wannamethee 2005). There is also some evidence that uric acid is an independent risk factor for stroke, diabetes, and metabolic syndrome (Dimitroula et al. 2008; Hayden and Tyagi 2004), but protects against Parkinson’s disease (Chen et al. 2009).

PFOA has been used in the manufacturing of fluoropolymers at a chemical plant in Washington, West Virginia, since 1951. In 2001, a group of residents from the Ohio and West Virginia communities in the vicinity of the Washington Works plant filed a class action lawsuit against the plant, alleging health damage due to contamination of human drinking water supplies with PFOA. The settlement of this lawsuit led to a baseline survey called the C8 Health Project. This survey was conducted in 2005–2006; data were gathered from 69,000 current and former residents of Ohio and West Virginia who had lived, worked, or attended school in six contaminated water districts surrounding the chemical plant. The C8 Health Project included blood draws and subsequent measurement of serum PFOA and serum PFOS, as well as clinical chemistries, including uric acid.

In the present study, we analyzed the data of adults ≥ 20 years of age to determine whether associations exist between PFOA or PFOS and uric acid in this population. The exposure metrics in this study are serum PFOA and PFOS measured in 2005–2006, and the outcome is concurrent uric acid level.

## Materials and Methods

### Study population

**Figure 1:**
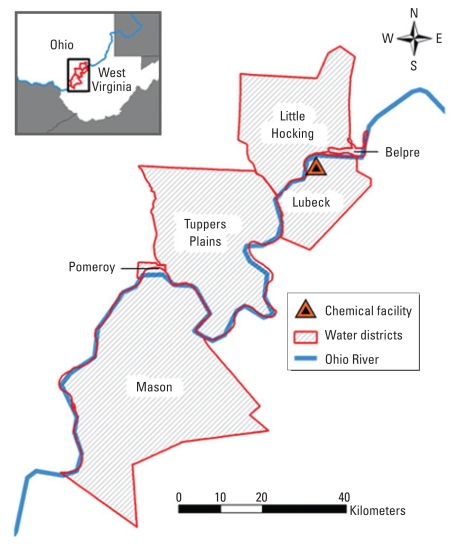
Six water districts contaminated with PFOA.

Study subjects participated in the C8 Health Project (Frisbee et al. 2009; Steenland et al. 2009a). The purpose of the C8 Health Project was to collect health data from residents covered under the legal settlement of a class action lawsuit, which included a battery of blood tests and measurement of serum levels of PFOA and PFOS. The C8 Health Project began in August 2005 and completed enrolling subjects in August 2006. Subjects were eligible to participate in the C8 Health Project if they had consumed public drinking water supplied by any of six contaminated water districts or from a small number of private wells known to be contaminated, and if they could provide documentation that they had lived, worked, or attended school in a contaminated water district for at least 1 year before 3 December 2004. The six water districts all had documented PFOA contamination of public water (≥ 0.05 ng/mL). Figure 1 shows the approximate boundaries of the six water districts. Subjects filled out an extensive questionnaire (Frisbee et al. 2009) and came to local survey stations to donate a blood sample.

The C8 Health Project collected data on 69,030 subjects; of these, 54,591 were ≥ 20 years of age and were included in our study. To estimate the participation rate among current residents in 2005–2006 among adults ≥ 20 years of age, we used census data. Estimates of the population residing in the six water districts were made based on population estimates for census block groups in 2005. Block groups are smaller than census tracts but larger than census blocks. To find the population of each water district, we determined which block groups were entirely within the water district. We then determined which block groups intersected the boundaries of the water districts. For those that intersected, we then calculated the ratio of water district area to block group area within each block group and multiplied the ratio by the block group population. We then summed the populations for the entire water district and then summed across all six water districts. Finally we determined the numbers of current residents (63% of total participants) in the water districts who participated in the C8 Health Project in 2005–2006, and divided these residents (33,001) by the population (40,721) to find a participation rate of 81% among current residents ≥ 20 years of age.

### Statistical analysis

All analyses were done using the SAS statistical package (version 10; SAS Institute Inc., Cary, NC). Analyses were conducted using linear regression with uric acid as the outcome. Residuals were checked for normality. The exposure variables were serum PFOA or PFOS. Most analyses used categorical exposure variables (deciles, lowest decile as reference), to avoid any assumptions about the shape of a parametric model, taking advantage of the large sample size that permitted adequately precise estimates across multiple categories. As a test for linear trend, we used the *p*-value of the parameters for PFOA or PFOS as continuous variables. We also conducted some analyses using the log transform of the exposure variables (PFOA and PFOS), because the log transform appeared to fit the data well. Covariates in the model were chosen a priori because of their established relationship to uric acid, such that they were potentially confounding variables. Covariates included age (18–39, 40–49, 50–59, 60–69, 70–79, ≥ 80 years), sex, body mass index (BMI), education as a measure of socioeconomic status (less than high school, high school, some college, college plus), smoking (never, current, former), current alcohol consumption, and serum creatinine as a marker of kidney function. The log of creatinine was used because it provided a better fit to the data (higher likelihood) than either untransformed continuous or categorical variables for creatinine. All covariates were statistically significant predictors of uric acid, in the predicted direction.

In addition to the linear regressions described above, we also ran logistic regression models for the dichotomous outcome hyperuricemia, which was defined as serum uric acid > 6 mg/dL for women and > 6.8 mg/dL for men (Johnson et al. 2003). In these models we used quintiles of PFOA (0–11.4, 11.5–20.6, 20.7–38.9, 39.1–88.6, ≥ 88.7 ng/mL) or PFOS (0–12.1, 12.2–17.4, 17.5–23.2, 23.2–31.8, ≥ 31.90 ng/mL). These analyses were adjusted for the same covariates used in linear regression.

We provided graphical representation of the linear regression results by showing the predicted trend in uric acid by decile of either PFOA or PFOS, given a covariate profile for an average subject. We used the median of each decile for graphing the *x*-axis.

### Laboratory methods

The analytical method for PFOA and PFOS used in this study has been described in detail (Flaherty et al. 2005; Longnecker et al. 2008). Both fluorocarbons are found in the serum fraction of the blood; they are not lipophilic (but rather proteinophilic), and no adjustment is made for lipid fractions. Briefly, the method uses liquid chromatography separation with detection by tandem mass spectrometry. Extraction of the serum samples was done using acetonitrile. Estimates of precision for PFOA were generally within ±10% for multiple replicates of individual samples over the range of 0.5–40 ng/mL with a more precise relative precision measure of approximately 1% for highly fortified (10,000 ng/mL) samples. Relative precision estimates for PFOS were similar. The limit of detection for both chemicals is 0.5 ng/mL. Less than 1% of values of each chemical were less than the limit of detection, and these were assigned 0.25 ng/mL.

Fasting blood samples were not required and were obtained at any time the participants came to the study site, that is, throughout the course of the day. Serum was separated from red cells and placed in transport tubes and refrigerated for shipment to the lab. Uric acid was measured in serum via the enzymatic uricase method. Uric acid is oxidized by uricase to allantoin and hydrogen peroxide. 3,5-Dichloro-2-hydroxybenzene sulfonate coupled with 4-aminoantipyrine and hydrogen peroxide, in the presence of peroxidase, forms a colored complex that is measured at 520 nm. The color intensity is proportional to the concentration of uric acid in the sample.

## Results

Table 1 provides descriptive statistics on uric acid, PFOA, PFOS, and covariates in the model. The distribution of PFOA among the study participants is more highly elevated and highly skewed than that of the general U.S. population, whereas the distribution of PFOS generally conforms to that expected based on the U.S. population. Uric acid levels conform to what would be expected in an adult population. There were few missing data, so the final model included 97% of the adult participants.

**Figure 2:**
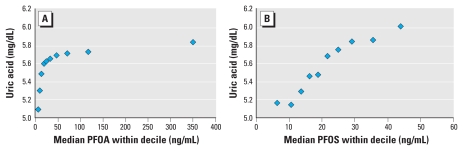
Observed uric acid with increasing PFOA (*A*) and PFOS (*B*) unadjusted for covariates.

**Figure 3:**
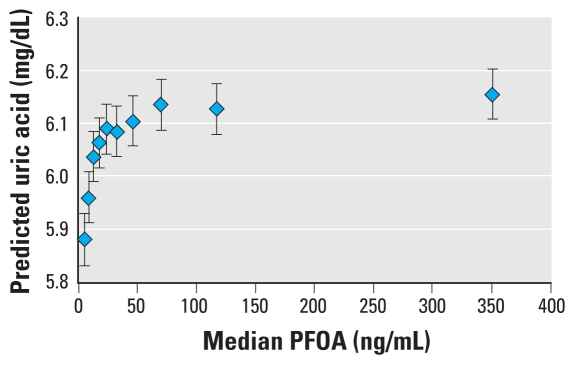
Predicted uric acid with increasing PFOA. Predicted value from regression model for an average participant: 45 years of age, 0.95 mg/dL creatinine, high school education, male, 28.55 kg/m^2^ BMI, nonsmoker, nondrinker. Data are population means and 95% CIs.

**Figure 4:**
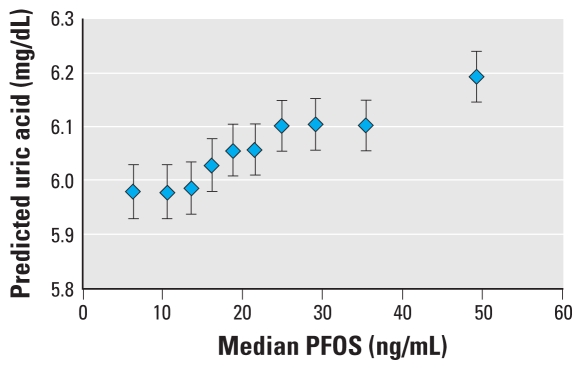
Predicted uric acid with increasing PFOS. Predicted value from regression model for an average participant: 45 years of age, 0.95 mg/dL creatinine, high school education, male, 28.55 kg/m^2^ BMI, nonsmoker, nondrinker. Data are population means and 95% CIs.

Table 2 shows results of the model for PFOA and uric acid, and Table 3 shows the results for PFOS and uric acid. A test for linear trend (using untransformed PFOA and PFOS) was highly significant (coefficient ± SE: PFOA, 0.00011 ± 0.00002; PFOS, 0.00070 ± 0.00006; *p* < 0.0001) in both cases. Figure 2 shows the actual observed mean values of uric acid with deciles of PFOA and PFOS, without covariate adjustment. Figures 3 and 4 display the model’s predicted values for uric acid by decile of PFOA and PFOS, respectively, adjusted for covariates. The model-predicted values have the same pattern as the observed data, although they are slightly higher because they are the predicted values for males, which have slightly higher levels than the entire population, which is the basis for Figure 2. Tables 2 and 3, and Figures 2–4, indicate a close to monotonic increase in uric acid with an increase in either PFOA or PFOS. There is an increase in uric acid of 0.2–0.3 μg/dL from the lowest to the highest decile for both PFOA and PFOS. The exposure–response curve for PFOA appears to tail off at the highest exposures, whereas for increasing PFOS, uric acid increases approximately linearly. The uppermost exposure levels of PFOA in this population far exceeded expected U.S. levels; this was not the case for PFOS.

PFOA and PFOS were correlated in our data (Spearman correlation coefficient, 0.31). When we included both PFOA and PFOS in the linear regression model, both variables continued to show a positive linear trend; the trend for PFOA was slightly diminished, but the one for PFOS was notably diminished. This finding suggests that of the two variables, PFOA was the more important. The trend for PFOA peaked at a 0.25 μg/dL increase for uric acid at the highest decile of PFOA (vs. 0.28 μg/dL without PFOS in the model), whereas the trend for PFOS peaked at 0.13 μg/dL uric acid increase for the highest decile of PFOA (vs. 0.22 μg/dL without PFOA in the model).

Analyses of hyperuricemia as an outcome (> 6.0 mg/dL for women, > 6.8 mg/dL for men) by quintile of PFOA yielded odds ratios (ORs) of 1.00, 1.33 (95% CI, 1.24–1.43), 1.35 (95% CI, 1.26–1.45), 1.47 (95% CI, 1.37–1.58), and 1.47 (95% CI, 1.37–1.58). The test for linear trend, via determining the statistical significance of the coefficient for a continuous variable PFOA, was significant [coefficient (SE), 0.00023 (0.0004); *p* < 0.0001], although the trend in OR appeared to plateau rather than increase in a strictly linear fashion. The corresponding ORs for PFOS quintiles were lower: 1.00, 1.02 (95% CI, 0.95–1.10), 1.11 (95% CI, 1.04–1.20), 1.19 (95% CI, 1.11–1.27), and 1.26 [95% CI, 1.17–1.35; test for trend, continuous variable PFOS, coefficient (SE), 0.0050 (0.0007); *p* < 0.0001]. When we entered both PFOA and PFOS into the logistic model for hyperuricemia, we observed the same pattern of results, with a slightly less steep trend (OR for top quintile: PFOA, 1.42; 95% CI, 1.32–1.53; PFOS, 1.13; 95% CI, 1.05–1.22). We observed no significant interaction between PFOA and PFOS.

Only 119 people (0.2%) had creatinine > 2.50 mg/dL, which suggests kidney disease that can affect uric acid; excluding these participants from the analyses did not change the results. A broader exclusion cut point of 1.50 mg/dL excluded 739 and again had no effect on results. Inclusion of a variable for taking cholesterol medication (15% took medication) was not a significant predictor of uric acid. Among those who were not taking such medication, we found that measured total cholesterol was a significant positive predictor of higher uric acid but had little effect on the ORs for PFOA or PFOS.

We found no significant interactions between PFOA and age, creatinine, or BMI. We found a significant interaction for sex; although both sexes had showed a significant positive exposure–response relation, it was more consistently linear for females than for males. For hyperuricemia, the ORs for females were 1.20 (95% CI, 1.08–1.33), 1.26 (95% CI, 1.13–1.41), 1.35 (95% CI, 1.21–1.51, and 1.42 (95% CI, 1.26–1.58), whereas for males the corresponding ORs were 1.31 (95% CI, 1.19–1.45), 1.26 (95% CI, 1.15–1.39), 1.37 (95% CI, 1.25–1.51), and 1.34 (95% CI, 1.22–1.48).

We also analyzed a subset of participants who had a PFOA level ≤ 20 ng/mL, dividing them into quartiles of PFOA exposure, so that the referent group had levels similar to those of the U.S. general population. Those with 5–9.9 ng/mL (*n* = 2,012), 10–14.9 ng/mL (*n* = 6,841), and 15–20 ng/mL (*n* = 7,387) had predicted increases in uric acid of 0.14 (95% CI, 0.07–0.20), 0.21 (95% CI, 0.15–0.27), and 0.24 mg/dL (95% CI, 0.18–0.31) above those with PFOA levels < 5 ng/mL.

## Discussion

We observed a positive association between PFOA and uric acid, although the absolute magnitude of the change in uric acid from lowest to highest decile was modest. We observed a significant positive association in two previous studies of workers exposed to high levels of PFOA. No details were given for one of these studies (Sakr et al. 2007a), whereas in the other study of 160 workers the exposure–response coefficient was about twice as high as our own (per unit PFOA) (Costa et al. 2009).

We also observed an elevated risk of hyperuricemia among subjects in the top quartile of PFOA. PFOS showed a similar relationship with uric acid as did PFOA, but with less pronounced trends. PFOA appeared to be more strongly associated with uric acid than was PFOS. The ORs for hyperuricemia were higher for PFOA than for PFOS (1.47 vs. 1.26 for the top quintile vs. the lowest quintile). Inclusion of both fluorocarbons in the hyperuricemia model only slightly diminished positive trends with PFOA but had more effect on trends with PFOS.

Serum PFOA levels were quite high overall in this community primarily due to contamination of drinking water (although 5% of the population had worked at the chemical plant and had high levels due to occupational exposure). However, a large number of people had low levels, similar to the U.S. population. Serum PFOS was present at levels similar to those of the U.S. population. Restriction of the data to those with lower levels of PFOA suggested that even slight increases in PFOA above background were associated with significant increases in uric acid.

In linear regression analyses, the exposure–response curve for PFOA and uric acid appeared to attenuate at the highest exposures, possibly reflecting saturation of a biological mechanism at high doses, whereas the curve for PFOS did not.

The percentage of the variance (change in *R*^2^) in uric acid attributable to PFOA (or PFOS) is small, only about 1%. However, many variables that are important predictors explain only a small amount of the variation of something else. For example, in our model predicting cholesterol in this population (Steenland et al. 2009b), removal of significant and well-established cholesterol predictors—exercise, smoking, sex, alcohol, and socioeconomic status combined—reduces the *R*^2^ of our model < 1% (the reduced model contains only BMI and age). Furthermore, if there is a causal relationship between PFOA and uric acid, we may have misclassified PFOA by using current levels, because past levels may be the more relevant predictor. This would bias our findings toward the null, decreasing the amount of variance explained.

A mechanism by which PFOA (or PFOS) might lead to higher uric acid is unknown. However, some data suggest that PFOA can induce oxidative stress in human liver cells (Panaretakis et al. 2001; Yao and Zhong 2005). It is in turn possible that such oxidative stress may be associated with increased uric acid (Patterson et al. 2003).

A second possible mechanism by which PFOA and uric acid might be associated without being causally linked is via shared renal transport transporters governing excretion of each substance. Organic ion transporters 1 and 3 (OAT 1 and 3) are involved in tubular secretion. OAT 1 and 3 have high affinity for PFOA (Nakagawa et al. 2008). Recent studies also show that OAT 1 and 3 are involved in urate secretion (Eraly et al. 2008). So it is possible that if the levels of PFOA increase, the secretion of urate is decreased and therefore blood urate levels may secondarily increase. However, whether this shared transporter hypothesis is relevant in humans remains speculative at this point.

Our findings are of interest because uric acid itself has been linked to hypertension and possibly other cardiovascular outcomes. A strength of our study is the large population and the fact that the participation rate in the community was high, lessening concern about chance findings and about potential selection biases. A limitation is that we did not have data on blood pressure for our population, making it impossible to directly assess a possible fluorocarbon–blood pressure relationship. Perhaps the principal limitation of our study is that, despite the associations we observed, causal inference is limited by the cross-sectional nature of the data. We cannot know whether the rise in PFOA or PFOS preceded the rise in uric acid. It is also possible that concentrations of both perfluorinated compounds rise with increased uric acid because all three are related to some other as yet unknown biological mechanism, an interpretation consistent with the parallel effects of both PFOA and PFOS, assuming this mechanism was related to perfluorinated compounds in general.

## Figures and Tables

**Table 1:** Descriptive statistics (*n* = 54,951) for adults ≥ 20 years of age in the Mid-Ohio Valley in 2005–2006.^a^

Variable	Mean ± SD (interquartile range)	Median	Percent
Continuous variables			
Uric acid (mg/dL)	5.58 ± 1.55 (4.5–6.6)	5.50	NA
PFOA (ng/mL)	86.4 ± 261.3 (13.5–71.4)	27.9	NA
PFOS (ng/mL)	23.4 ± 16.1 (13.6–29.3)	20.2	NA
Age (years)	45.0 ± 15.9 (33–57)	44.0	NA
BMI	28.7 ± 6.5 (24.2–32.0)	27.5	NA
Creatinine (mg/dL)	0.95 ± 0.28 (0.8–1.1)	0.90	NA

Categorical variables used in model			
BMI			
≤ 24	21.5 ± 1.7 (19.1–23.7)	21.9	25
24–27.4	25.8 ± 1.0 (25.0–26.6)	25.8	25
27.5–31.9	29.5 ± 1.26 (28.4–30.6)	29.4	25
≥ 31.9	37.3 ± 6.35 (33.5–39.5)	35.6	25
Age (years)			
18–39	28.6 ± 6.42 (23–34)	29	39.8
40–49	44.8 ± 2.9 (42–47)	45	21.0
50–59	54.3 ± 2.9 (52–57)	54	18.7
60–69	64.1 ± 2.8 (62–66)	64	12.3
70–79	73.7 ± 2.8 (71–78)	73	6.2
≥ 80	83.8 ± 3.7 (81–86)	83	1.9
Smoking status			
Never smoker	NA	NA	48
Current smoker	NA	NA	26
Former smoker	NA	NA	26
Current alcohol	NA	NA	48
Male	NA	NA	48
Education			
< High school	NA	NA	13
High school	NA	NA	42
Some college	NA	NA	32
≥ College	NA	NA	18
High uric acid (> 6.0 mg/dL for women, > 6.8 mg/dL for men)	NA	NA	24

NA, not applicable.

a Maximum percentage of missing any variable was 1.1%.

**Table 2:** Results from model with PFOA and uric acid.^a^

PFOA (ng/mL)	Estimate	SE
0–7.8	0	
7.9–11.4	0.09	0.02
11.5–15.4	0.16	0.02
15.5–20.6	0.18	0.02
20.6–27.8	0.21	0.02
27.9–38.9	0.21	0.02
39.0–56.9	0.22	0.02
57.0–88.6	0.22	0.02
88.7–188.6	0.25	0.02
≥ 188.7	0.28	0.02

a Model *R*^2^ = 0.40, *n* = 53,458 (3% of data was lost because of missing values). Adjusted for age, creatinine, sex, smoking, education, BMI, and current alcohol consumption. All covariates are significant at *p* < 0.0001 in expected directions.

**Table 3:** Results from model with PFOS and uric acid.^a^

PFOS (ng/mL)	Estimate	SE
0–9.0 (referent)	0	
9.1–12.1	0.00	0.02
12.2–14.9	0.01	0.02
15.0–17.4	0.06	0.02
17.5–20.1	0.08	0.02
20.2–23.1	0.09	0.02
23.1–26.8	0.12	0.02
26.9–31.8	0.12	0.02
31.9–40.4	0.12	0.02
≥ 40.5	0.22	0.02

a Model *R*^2^ = 0.40, *n* = 53,458 (3% of data lost because of missing values). Adjusted for age, creatinine, sex, smoking, education, BMI, and current alcohol consumption. All covariates are significant at *p* < 0.0001 in expected directions.
